# Genetic architecture of gene expression in ovine skeletal muscle

**DOI:** 10.1186/1471-2164-12-607

**Published:** 2011-12-15

**Authors:** Lisette JA Kogelman, Keren Byrne, Tony Vuocolo, Nathan S Watson-Haigh, Haja N Kadarmideen, James W Kijas, Hutton V Oddy, Graham E Gardner, Cedric Gondro, Ross L Tellam

**Affiliations:** 1 CSIRO Livestock Industries, ATSIP, PMB CSIRO Aitkenvale, Townsville QLD 4814, Australia; 2 Wageningen University and Research Centre (WUR), Animal Breeding and Genetics, Wageningen, The Netherlands; 3 CSIRO Livestock Industries, Queensland Bioscience Precinct, St. Lucia, Brisbane, QLD 4067, Australia; 4 Division of Genetics and Bioinformatics, Faculty of Life Sciences, University of Copenhagen, 1870 Frederiksberg C, Denmark; 5 School of Environmental and Rural Science, University of New England, Armidale, NSW 2351, Australia; 6 School of Veterinary and Biomedical Sciences, Murdoch University, Murdoch, WA 6150, Australia; 7 The Australian Wine Research Institute, P.O. Box 197, Glen Osmond, SA 5064, Australia

## Abstract

**Background:**

In livestock populations the genetic contribution to muscling is intensively monitored in the progeny of industry sires and used as a tool in selective breeding programs. The genes and pathways conferring this genetic merit are largely undefined. Genetic variation within a population has potential, amongst other mechanisms, to alter gene expression via cis- or trans-acting mechanisms in a manner that impacts the functional activities of specific pathways that contribute to muscling traits. By integrating sire-based genetic merit information for a muscling trait with progeny-based gene expression data we directly tested the hypothesis that there is genetic structure in the gene expression program in ovine skeletal muscle.

**Results:**

The genetic performance of six sires for a well defined muscling trait, longissimus lumborum muscle depth, was measured using extensive progeny testing and expressed as an Estimated Breeding Value by comparison with contemporary sires. Microarray gene expression data were obtained for longissimus lumborum samples taken from forty progeny of the six sires (4-8 progeny/sire). Initial unsupervised hierarchical clustering analysis revealed strong genetic architecture to the gene expression data, which also discriminated the sire-based Estimated Breeding Value for the trait. An integrated systems biology approach was then used to identify the major functional pathways contributing to the genetics of enhanced muscling by using both Estimated Breeding Value weighted gene co-expression network analysis and a differential gene co-expression network analysis. The modules of genes revealed by these analyses were enriched for a number of functional terms summarised as muscle sarcomere organisation and development, protein catabolism (proteosome), RNA processing, mitochondrial function and transcriptional regulation.

**Conclusions:**

This study has revealed strong genetic structure in the gene expression program within ovine longissimus lumborum muscle. The balance between muscle protein synthesis, at the levels of both transcription and translation control, and protein catabolism mediated by regulated proteolysis is likely to be the primary determinant of the genetic merit for the muscling trait in this sheep population. There is also evidence that high genetic merit for muscling is associated with a fibre type shift toward fast glycolytic fibres. This study provides insight into mechanisms, presumably subject to strong artificial selection, that underpin enhanced muscling in sheep populations.

## Background

The genetic contribution to complex traits such as muscling in livestock production animals is often intensively monitored. The information is used as a tool in selective breeding programs, based on the relative genetic performances of sires, to enhance desirable traits within the population. This sire-based genetic information for a complex trait, which is typically the result of polygenic effects, is generated by extensive progeny testing for the trait [1]. The molecular mechanisms underpinning this genetic merit within a population are typically undefined but are likely to arise from polygenic changes in gene transcription mediated by genetic variation in promoters, transcriptional regulatory elements such as enhancers and insulators, and mRNA splicing sites as well as variants affecting mRNA turnover. Although there are several additional mechanisms that could impact phenotype independent of gene transcription including changes in protein intrinsic activity and mRNA translational efficiency, it has been suggested that polygenic transcriptional changes are probably the major influence on variation in complex traits within a population [2]. Genetic variation could alter gene expression via cis- or trans-acting mechanisms in a manner that impacts the functional activities of specific pathways that directly contribute to the trait [3]. The identification of these pathways has been an important goal of mammalian genomics.

One of the major advantages of using livestock to investigate the genetic and biological bases of complex traits is that the industry holds comprehensive genetic performance data for sires based on quantitative trait measurements made from large numbers of their progeny. The Poll Dorset breed of sheep has been intensively bred for rapid growth rate and superior muscling characteristics [1]. In particular, there is strong emphasis in breeding programs for the trait Eye Muscle Depth (EMD), which is measured ultrasonically in longissimus lumborum muscle at the 'C' site (45 mm from the centre of the spine) between the 12^th ^and 13^th ^rib in the progeny of breeding sires. Estimated breeding values (EBVs; a measure of relative genetic performance compared with a contemporary group) for this trait have been determined for many sires. Hence, sires with varying genetic performances for this trait can be identified from industry databases.

By integrating sire-based EBV information with progeny-based gene expression data obtained by microarray analysis we directly tested the hypothesis that there is genetic structure to gene expression in ovine skeletal muscle. Moreover, we employed an integrated systems biology approach using both an EBV weighted gene co-expression network analysis (WGCNA) and a differential gene co-expression network analysis to identify highly connected genes correlated with the trait and differentially connected genes in the high and low EBV sire groups, thereby identifying major functional pathways contributing to the genetics of enhanced muscling [4-6].

## Results and Discussion

### The genetics of Eye Muscle Depth in sheep

Six Poll Dorset sires with extensive progeny records were selected on the basis of having a range of EBVs for the Eye Muscle Depth (EMD) (Table 1). EMD is a skeletal muscling trait measured by ultrasound at a predefined site in longissimus lumborum skeletal muscle in yearlings. The EBVs are predictions of the sire's relative genetic merit for the EMD trait based on progeny performance data collected from large numbers of animals over two seasons (2003/2004 and 2004/2005). Positive and negative EBVs indicate sire genetic performance better or worse than the industry wide average set for a baseline population in 2001. Three sires (sires I, II and III) were in the top 1^st ^to 15^th ^percentiles for all industry sires for this trait (high muscling group) and two sires (sires IV and VI), each with EBVs of -1.07 were in the 95^th ^percentile (low muscling group), while one sire (sire V) had an EBV of +0.9 (45^th ^percentile). By contrast with the sire based percentile rankings for the high muscling group, sire V was included in the low muscling sire group. A total of 40 half sib progeny derived from the six sires were selected for microarray analysis using RNA extracted from longissimus lumborum skeletal muscle. The number of progeny sampled for each sire is shown in Table 1.

**Table 1:** The genetics of Eye Muscle Depth (EMD) in Poll Dorset sheep

Sire	**EMD EBV**^**1,2**^	**Accuracy**^**3**^ (%)	Percentile **Ranking**^**4**^	Muscling Group	Progeny ID	Progeny
I	2.95	93	1	High	1-7	7
II	1.49	97	15	High	8-11	4
III	1.78	98	10	High	12-19	8
IV	-1.07	97	95	Low	20-27	8
V	0.90	90	45	Low^5^	28-35	8
VI	-1.07	93	95	Low	36-40	5

^1^Estimated Breeding Value of a sire for the yearling Eye Muscle Depth (EMD) trait measured by progeny testing. EBVs were derived from data extracted from LAMBPLAN [45].

^2^EMD EBV (mm) is a measure of the difference in the trait from the industry-wide contemporary average set in 2001.

^3^Accuracy reflects the number of progeny with phenotype information used to calculate the EBV. Accuracy ranges from 0-99% and indicates the probability of an EBV changing with the addition of more progeny data. The magnitude of possible change decreases as accuracy increases. Accuracy below 75% is regarded as low, between 76-90% as medium and above 90% as high.

^4^Percentile ranking of the sire amongst contemporaries for the EMD trait.

^5^Sire group V was classed in the low muscling sire group because of its percentile ranking relative to sires in the high muscling group.

### Microarray gene expression analysis of skeletal muscle samples

**Figure 1:**
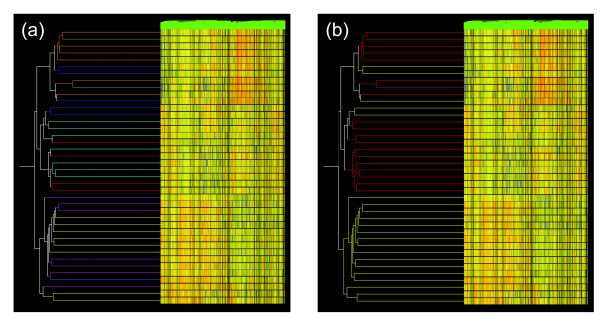
**Hierarchical cluster analysis showing the genetic architecture of gene expression in skeletal muscle**. Unsupervised hierarchical cluster dendrogram of gene expression was undertaken using 40 skeletal muscle samples derived from the progeny of six sires characterised as having high or low EBVs for the trait EMD (Eye Muscle Depth). Clustering was undertaken by using GeneSpring GX software (Agilent Technologies) with selection for "condition tree" and "gene tree" with "Standard Correlation". Rows represent individual animals while columns represent genes. Different colours in the heat map represent gradients of gene expression levels: red, higher expression; blue, lower expression; yellow, no difference in gene expression. The branch lengths indicate the correlation with which samples were joined, with longer branches indicating a lower correlation. Individual genes could not be resolved at this magnification. (a) Branch length coloured for sire group. Sire group I, light blue; II, red; III, brown; IV, yellow; V, purple; VI, dark blue (the colours do not correspond with any of those used in subsequent network analyses). (b) Branch length coloured for sire EBV for EMD: red, high EBV group; yellow, low EBV group.

The bovine Affymetrix microarray was used to measure gene expression in muscle samples from each of 40 progeny produced from the six selected sires. Previous studies have extensively validated the use of this microarray with ovine samples, although there is some data loss due to species specific differences in probe set sequences [7-9]. The microarray data were initially processed by using the Affymetrix processing software MAS5 and significantly expressed genes were subsequently filtered for the independent MAS5 flag calls of *present *or *marginal *in all 40 microarrays thereby yielding 5394 probes. This was a highly conservative strategy designed to select for genes that were transcribed in all of the muscle samples. These data were then used as input for unsupervised hierarchical clustering analysis. Figure 1 shows the hierarchical cluster analysis, which used signals from the 5394 genes in each of the 40 samples. There was strong association of gene expression with sire group (Figure 1(a)) (P = 1.30E-8; Fischer's Exact Test). Moreover, the data also showed strong correlation with sire EBV status (Figure 1(b)) (P = 7.6E-12; Fischers Exact Test). Thus, there was substantial genetic structure to the gene expression program in the progeny skeletal muscle samples suggesting that this directly contributed to the EBV status of the sires. The origins of this effect could reflect the impact of genetic variation on gene expression at the time of sampling or involve changes at earlier developmental times. One trivial alternative possibility is that this program simply reflected a sire-based genetic effect independent of direct contribution to the muscling EBV status. This is unlikely as the tissue examined for gene expression was the same as that measured to establish the EBV status of the sires. In addition, analyses of enriched gene functions and pathways detailed below were highly relevant to a muscling phenotype. There is no a priori reason why this would occur if these results were due to an indirect sire based genetic effect. The gene expression profile of one sire group (VI; coloured dark blue in Figure 1(a)) proved an exception as it was not consistent with the EBV status of the sire in the context of the results from the other sire groups. The progeny of this sire showed much greater variability in gene expression compared with other sire groups (results not shown).

To further dissect this gene expression information a systems biology approach using gene networks was subsequently employed.

### Gene expression network construction

The architecture of gene expression networks may provide insight into the biological mechanisms underpinning the genetics of the EMD trait. A gene co-expression network analysis relies on the hypothesis that a strong correlation in mRNA expression levels for a group of genes implies that these genes work cooperatively in contributing to a trait. The degree of connectivity of a gene in the network is simply the number of other genes that show correlated expression with that gene.

**Figure 2:**
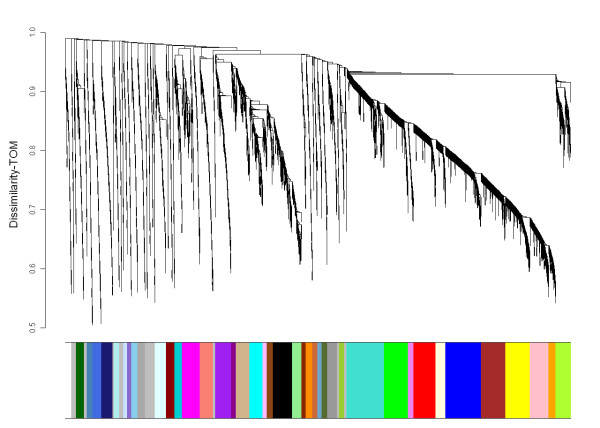
**Hierarchical clustering dendrogram of the Topological Overlap Measure (TOM) matrix for the gene expression data**. The topological overlap measure plot shows clusters of highly interconnected genes (modules). Genes were assigned to modules named by the colours below the dendrogram using the static tree cutting method. A total of 42 modules each containing more than 40 genes was used as input for the subsequent WGCNA and the differential coexpression network analyses.

A component of the weighted gene co-expression network (WGCNA) approach [5,10] was initially employed to construct the network. This approach has been widely employed to build a weighted gene co-expression network based on gene expression and the absolute Pearson correlation coefficient between gene expression levels to detect clusters of genes correlated with a trait [4,11-13]. The GC-RMA microarray data processing algorithm was used to generate the primary gene expression data input for gene network construction. The 3,500 most connected genes (genes showing correlated expression with many other genes) in each of the high and low muscling datasets (5,223 unique genes in the combined set) were employed for initial network construction using the WGCNA package, which is a connectivity based method that detects clusters of highly interconnected genes based on soft thresholding using a power function and scale free topology [5]. The adjacency matrix was calculated by raising the correlation matrix of gene expression profiles to a power β, which was chosen based on the scale-free topology criterion. A β value of 4 was chosen (R^2 ^= 0.8). Modules of genes were defined using the topological overlap measure (TOM), which shows the degree of overlap in shared neighbours between pairs of genes in the network [14]. The TOM-plot visualized this relative interconnectedness between pairs of genes and showed how modules (clusters of highly interconnected genes) were created. Modules were defined using the Dynamic Tree Cutting algorithm on a dendrogram created from the dissimilarity-TOM matrix, which was calculated using the adjacency matrix [15]. This construction led to the identification of 42 modules, with each containing more than 40 genes (Figure 2). These modules were then used for the WGCNA and the differential network analyses. Each module was uniquely identified by a colour name.

### Weighted gene co-expression network analysis (WGCNA)

The identified modules were then selected on the basis of their module correlation (MC), which is the absolute correlation between the module eigengene (a representative gene expression profile for the module), and sire EBV for EMD. Further, genes in the identified modules were then retained based on their intra-modular connectivity (a single gene measure of connectivity within a module) and the absolute correlation of the gene expression with the sire EBV for EMD. Four modules were identified: Module Violet_WGCNA _(MC = 0.54, 39 genes), Module Cyan_WGCNA _(MC = -0.52, 88 genes), Module Tan_WGCNA _(MC = -0.52, 33 genes) and Module Lightgreen_WGCNA _(MC = -0.42, 42 genes). Additional File 1 lists the genes present in these four WGCNA modules.

**Figure 3:**
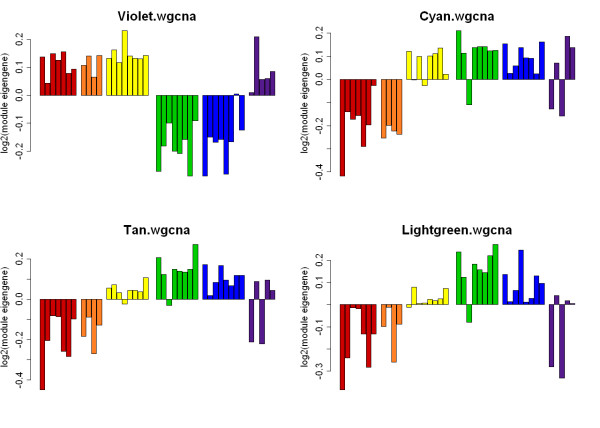
**Sire group expression profiles of eigengenes for the identified WGCNA modules**. Four WGCNA modules were selected on the basis of their module correlation (MC), which is the absolute correlation between the module eigengene and the quantitative EBV status. Genes in these modules were then retained based on their intra-modular connectivity (a single gene measure of connectivity within a module) and the absolute correlation of the gene expression with the EBV for EMD. Module Violet **_WGCNA _**(MC = 0.54, 39 genes), Module Cyan **_WGCNA _**(MC = -0.52, 88 genes), Module Tan **_WGCNA _**(MC = -0.52, 33 genes) and Module Lightgreen **_WGCNA _**(MC = -0.42, 42 genes). The eigengene expression of each sire group is uniquely coloured. Sire group I (red; high EBV); sire group II (orange, high EBV); sire group III (yellow, high EBV); sire group IV (green, low EBV); sire group V (blue, low EBV); sire group VI (purple, low EBV). Each bar within a sire group represents a single animal. There is no relationship between sire group colour and module colour name.

Figure 3 shows the expression profiles of the module eigengenes for the four identified WGCNA modules for each sire group. The module eigengene is a representative gene expression profile that summarises the expression profiles of all genes in the module. The first three sire groups (I-III) had high EBVs for EMD while the last three (IV-XI) represented the lower muscling sire groups. In all four WGCNA modules the relative trends for the module eigengenes were coordinate in sire groups I, II, IV and V suggesting functional interactions of the genes within these modules. Of particular note was the Violet_WGCNA _module, which was the most different by comparison with other modules. In this module there was strong correspondence between sire module eigengenes and high muscling EBV status. The sire group III eigengenes were not coordinate with the other sire groups in all modules. The reasons for this are not clear but could reflect different mechanisms underpinning the high EBV status for this sire group. Sire group VI, which corresponded with the atypical sire group revealed in Figure 1, generally showed variation in the direction of the module eigengene relative to other sire groups and considerable variation even within the sire group, unlike the others. This may indicate that genes contributing to EBV status may be segregating within this sire group and therefore generating a greater range in progeny performance data. Overall, these results again demonstrate strong genetic architecture in gene expression as a function of both sire group and EBV status.

### Functional enrichment analysis of genes in WGCNA modules

Functional enrichment analysis was used to assign biological relevance to the genes present in each of the four WGCNA modules in the context of module eigengenes for the sire groups. These analyses used AgriGO [16], the Database for Annotation, Visualization and Integrated Discovery (DAVID) [17,18]) or Gene Set Enrichment Analysis (GSEA) [19]. While there is overlap in the functionalities of these databases, each also has unique capabilities.

**Figure 4:**
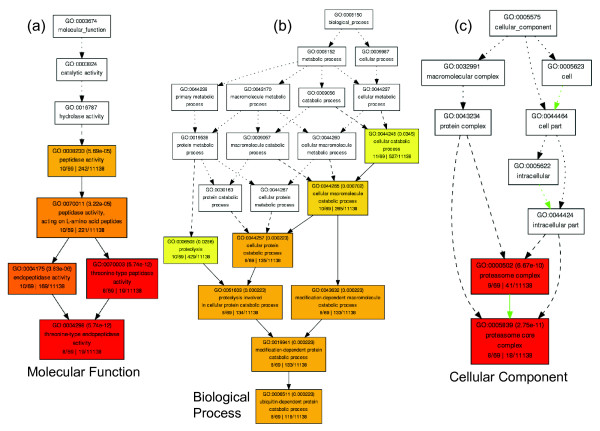
**Hierarchical tree graphs of over-represented GO terms for genes in the Cyan**_**WGCNA **_**module**. Hierarchical tree graphs of over-represented gene ontology (GO) terms for genes in the Cyan **_WGCNA _**module were constructed using AgriGO [16]. Boxes in the graphs represent GO terms labelled by GO number, term definition and statistical information. Significant terms (adjusted P ≤ 0.05) are coloured. The degree of colour saturation of a box is positively correlated to the enrichment level of the term. Solid, dashed, and dotted lines represent two, one and zero enriched terms at both ends connected by the line. GO categories: (a) Molecular Function; (b) Biological Process; (c) Cellular Component.

Genes present in the Cyan_WGCNA _module were strongly enriched for GO terms associated with protein catabolism mediated by the *proteosomal components*, *ubiquitin mediated proteolysis *and *threonine endopeptidases *(AgriGO analysis; Figure 4). This was also apparent from analysis of individual functional categories using DAVID e.g. KEGG pathway (*proteosome*; P = 2.16E-9) and INTERPRO Protein Domain (*proteosome alpha subunit*; P = 1.42E-10). The proteosome consists of a highly ordered macromolecular complex of multicatalytic proteases which function in energy dependent regulated proteolysis. Before a protein is degraded, it is first flagged for destruction by the ubiquitin conjugation system, which ultimately results in the attachment of a polyubiquitin chain to the target protein. The proteasome's regulatory cap binds the polyubiquitin chain, unfolds the protein, and feeds the protein into the proteasome's proteolytic core for destruction. Additional File 2 shows a table listing enriched clusters of terms using the DAVID functional annotation clustering approach, which gives a higher level perspective of functional term enrichments. The highest ranked cluster, which contained 26 terms, had an enrichment score of 6.88 (-log (geometric mean of the enriched term P-values)) and was represented by terms relating to the *proteosome*, *ubiquitin mediated proteolysis *and *proteolysis*. A second related cluster (enrichment score = 1.24) continued the focus on regulation of protein metabolism.

The positive eigengenes for the three low muscling sire groups in the Cyan_WGCNA _module suggest increased emphasis on protein catabolism in these groups. Consistent with this conclusion, previous studies of muscle deposition in sheep divergently selected for yearling growth rate over ten generations indentified enhanced protein degradation in hind-limb muscle in lines of these animals selected for low growth [20]. Conversely, the negative eigengenes for two of the high muscling sire groups in this module suggest decreased emphasis on protein catabolism, which is consistent with more skeletal muscle protein in these two groups. Next, the genes within the Cyan_WGCNA _module were examined by using GSEA for enrichment of conserved cis-acting regulatory motifs. This database included conserved gene-associated anonymous motifs in the human, mouse, rat and dog genomes and conserved gene-associated transcription factor binding motifs. The conserved motifs were restricted to a 'promoter' sequence window corresponding to ± 2 kb of the transcription start site. Additional File 3 shows that this module was associated with four enriched transcription factor motifs (binding sites for NFE2L1 (nuclear factor erythroid 2-related factor 1; confusingly sometimes called NRF1), NRF1 (nuclear respiratory factor 1), GABPB2 (GA-binding protein subunit beta-1) and one anonymous motif), which may participate in limiting muscle formation in the low EBV sire group. Interestingly, NRF1 induces expression of the key promyogenic transcription factor MEF2A (myocyte-specific enhancer factor 2 A) and has roles in regulating mitochondrial biogenesis and proteosome synthesis [21,22]. The enrichment for this transcription factor motif in the genes present in this module is consistent with a dual functional role for NRF1 in potentially balancing myogenesis and mitochondria production with energy dependent protein catabolism. It may also be speculated that in the high muscling sire groups represented by sire groups I and II, there is decreased emphasis on mitochondria biogenesis, which could reflect a fibre type shift toward fast twitch glycolytic fibres. These fibres are known to contain fewer mitochondria than slow twitch oxidative fibres [23].

Module Tan_WGCNA _was enriched for terms associated with *RNA processing *as defined by DAVID functional annotation clustering (enrichment score = 2.53; Additional File 2). Consistent with this, analysis of the KEGG pathway showed enrichment for the *splicosome *(P = 0.03). The former analysis also revealed a relatively small cluster enriched for terms associated with the cytoskeleton but this was below the threshold for cluster significance (data not shown). AgriGO analysis confirmed enrichment for *cytoskeleton protein binding *(P = 0.007) in the GO Molecular Function category but there were no other significant GO terms in the Biological Process and Cellular Component GO categories (results not shown). The eigengene sire group pattern for this module is similar to that of the Cyan_WGCNA _module. Thus, there was increased emphasis on RNA turnover and cytoskeletal reorganisation in the low muscling sire groups. A single transcription factor binding motif (GABPA; GA-binding protein alpha chain) was enriched in promoters of genes in this module (Additional File 3). This transcription factor is also involved in nuclear control of mitochondria function [24].

Functional terms representing protein synthesis at the level of *ribosome protein function *(KEGG Pathway; P = 1.22E-29) were strongly enriched in Module Lightgreen_WGCNA_. Additional File 4 shows AgriGO hierarchical tree graphs for each of the GO categories, which are consistent with this conclusion. DAVID functional annotation clustering not only revealed over-representation of ribosome function in this module (enrichment score = 20) but additionally suggested that mitochondrial function was also enriched (enrichment score = 1) (Additional File 2). The sire group eigengenes for the Lightgreen_WGCNA _module indicate that the expression of genes encoding ribosome proteins is positively correlated with the low muscling group. One possible explanation for this finding is that in the low muscling animals there is increased emphasis on enhancement of the ribosome protein machinery as compensation for constraints on muscle formation at the levels of myogenesis or myofibril hypertrophy responses. The enrichment for terms associated with mitochondrial function in this module is consistent with shifts toward slow twitch oxidative fibres in the low muscling group and fast twitch glycolytic fibres with lower mitochondrial content in the high EBV sire group. GSEA analysis indicated enrichment in this module for an anonymous promoter motif and the motif for binding by the ELK1 transcription factor (E twenty-six (ETS)-like transcription factor 1) (Additional File 3). The latter protein binds KLF4 (Krueppel-like factor 4) and acts cooperatively with histone deacetylases to suppress smooth muscle cell differentiation and postnatal growth [25,26]. This therefore suggests a transcription factor mediated regulatory mechanism which impacts both muscle cell formation and general protein synthesis in the low muscling group.

Module Violet_WGCNA _initially did not achieve significance for any functional terms. However, the striking relationship between this module and sire group EBV status (with the exception of sire group 6; Figure 3) suggested that further analysis was warranted. Consequently, AgriGO functional analysis was performed using less stringent parameters (P < 0.1 and ≥ 2 genes/term). This analysis identified *muscle sarcomere organisation *(p = 0.02) and *muscle development *(P = 0.02) in the GO category Biological Process while several terms relating to skeletal muscle fibre structure (*contractile fibre*, *myofibril*, *sarcomere*) were revealed in the Cellular Component category (Additional File 5). These data were consistent with coordinate emphasis on muscle structural components in the high muscling sire group. GSEA analysis identified three anonymous motifs and four motifs corresponding to known transcription factor binding sites (MEIS1 (homeobox protein Meis1), TGIF (TGFB-induced factor homeobox 1), RUNX1 (runt-related transcription factor 1) and TITF (thyroid transcription factor 1)) (Additional File 3). MEIS1 has been implicated in regulation of myogenesis through its control of the transcription of MYOD1 (myogenic differentiation 1), a pivotal promyogenic transcription factor and myostatin, a negative regulator of myogenesis [27,28]. RUNX1 has been shown to be involved in the maintenance of skeletal muscle [29]. Thus, increased muscle protein synthesis driven by myogenesis is probably a significant contributor to the high muscling sire groups.

The Violet_WGCNA _module contained a number of notable genes in addition to those encoding proteins directly contributing to skeletal muscle fibre structure. *ATP2A1 *encodes a Ca^2+^-ATPase that facilitates the ATP coupled translocation of calcium from the cytosol to the sarcoplasmic reticulum, and is intimately involved in muscle excitation and contraction. This protein isoform characterises fast twitch glycolytic muscle fibres (also known as type IIb), which contain fewer mitochondria than oxidative fibres [23,30]. This is consistent with decreased emphasis on mitochondria in the high muscling sire group as already suggested from analyses of the Lightgreen_WGCNA_, Tan_WGCNA _and Cyan_WGCNA _modules. The Violet_WGCNA _module also contained two additional genes whose encoded proteins, RYR1 (ryanodine receptor) and SRL (sarcalumenin), are also actively involved in calcium transport and storage in the sarcoplasmic reticulum. Collectively, this information suggests increased emphasis on sarcoplasmic reticulum function in the high muscling sire groups. This organelle controls the dynamics of calcium release and uptake during muscle contraction and is essential for normal muscle function [31]. Calcineurin A alpha (*PPP3CA*), another gene in this module, has key regulatory roles in skeletal muscle development, regeneration and hypertrophy and is therefore consistent with higher muscling occurring in sire groups I-III [32]. The module also contained *DICER1*, which encodes an important enzyme required for miRNA maturation. Several miRNA are known to be positive regulators of myogenesis [33,34] and DICER1 is essential for muscle development [35]. Interestingly, a simple binary comparison between the high and low muscling sire groups demonstrated that *DICER *was one of the most up-regulated genes (5.2 fold; adjusted P = 1.68e-5; result not shown). Enhanced DICER1 activity could lead to increased levels of mature miRNA thereby promoting increased myogenesis in the high muscling sire groups. The identification of several genes within this module that are known to regulate skeletal muscle formation confirms that the gene expression network analysis using the WGCNA approach provides valuable insight into the pathways regulating muscling which contribute to the muscling EBV.

### Differential co-expression network analysis

Another independent method to analyse these data used a differential co-expression network approach (CoXpress) which identified modules that were differentially co-expressed i.e. network modules containing genes that were highly correlated with the high muscling trait but not the low muscling trait and visa versa [6]. The hypothesis being tested was that the extremes of the muscling EBV trait were the result of changes in emphasis on specific biological pathways. Specific differentially connected modules were identified by comparing the behaviour of the 42 gene network modules for the high and low muscling EBV trait. A module was defined as differentially co-expressed when it was significantly different from random in one condition but not in the other condition. The conditions were high and low EBV status for EMD. The approach uses a resampling method to calculate a P-value for each module. Genes in the modules were then retained based on different criteria than for WGCNA and hence the gene contents of the identified modules were not identical when comparing this analysis with the WGCNA method. Genes in the differentially co-expressed modules were retained only if: (i) the intra-modular connectivity was > 0.6, and; (ii) the intra-modular connectivity with other modules was < 0.6. Five of the originally identified 42 modules were differentially co-expressed (Table 2). One module was found to be non-random in the high muscling group and random in the low muscling group (Violet_Diff_) and vice versa for the remaining four modules (Lightgreen_Diff_, Salmon_Diff_, Greenyellow_Diff _and Orange_Diff_). Thus, the genes in the Violet_diff _module may play a direct role in enhancing muscle development as they were highly connected in the high muscling sire group and correlated with EBVs for this group but not in the low muscling group. The remaining four modules were highly connected in the low muscling sire groups suggesting that the genes in these modules represented regulatory networks suppressing muscle formation and maintenance. The genes present in these modules are listed in Additional File 1.

**Table 2:** Differentially co-expressed modules defined by CoXpress^1^

Module	**Gene no**.	**P-value**^**2**^	**P-value**^**2**^	Mean correlation	Mean correlation	Mean correlation difference
		**LM**	**HM**	**LM**	**HM**	**HM - LM**

Violet_Diff_	51	0.61	0.00	0.03	0.59	+ 0.56
Lightgreen_Diff_	103	0.00	0.66	0.39	0.02	- 0.37
Salmon_Diff_	126	0.00	0.77	0.37	0.01	- 0.36
Greenyellow_Diff_	141	0.00	0.88	0.30	0.03	- 0.27
Orange_Diff_	74	0.02	1.00	0.27	0.01	- 0.26

^1^A module was differentially co-expressed when the pairwise correlations were not random in one condition (p < 0.05) and random in the other condition (p > 0.3) [6]. The conditions were high or low sire EBV status for EMD. HM, high muscling EBV; LM, low muscling EBV.

^2^The P-value is a measure of the significance that a module differs from random.

### Functional enrichment analysis of genes in differentially co-expressed modules

AgriGO analysis of the genes present in the Violet_Diff _module identified enrichment for *contractile fibre *and *myofibril *in the GO Cellular Component category (Additional File 6). These functional term enrichments are consistent with the high muscling EBVs being driven by increased muscle protein synthesis. DAVID functional annotation clustering identified aspects of *endonuclease activity*, *protein catabolism *and *vesicle mediated transport *as enriched although they were only weakly significant (enrichment scores 1.73, 1.20 and 1.12, respectively) (Additional File 7). In contrast with the AgriGO analysis, muscle structural components did not reach significance in the DAVID annotation clustering analysis. The reasons for this are not clear but are likely due to differences in inherent methodologies and significance thresholds (the high stringency option was used in the DAVID analysis). GSEA analysis identified five anonymous motifs and four motifs corresponding to transcription factor binding sites in the promoters of genes in this module (Additional File 3). Of the latter it is noted that C/EBP (CCAAT/enhancer-binding protein alpha) is essential for establishment and maintenance of energy homeostasis in neonates and it has a strong role in the development of adipose tissue [36]. One possibility is that there is cross-talk between regulatory mechanisms that govern muscle formation and adipose tissues in these animals. Motifs for TGIF, MEIS1 and RUNX1 binding were also identified in this analysis, as for the Violet_WGCNA _module.

The Light-green_Diff _module, as for the WGCNA analysis, was strongly enriched for *ribosome protein function *(KEGG Pathway; P = 1.32E-79) (Additional Files 7 and 8; DAVID functional annotation clustering and AgriGO analysis, respectively). There was also enrichment for *mitochondrial function*. This differentially expressed module was only significant for the low muscling EBV category and therefore it was consistent with previous conclusions that the low muscling EBV trait was associated with increased emphasis on protein translation, perhaps as a compensatory mechanism, and increased mitochondria formation or function. GSEA analysis confirmed enrichment for the ELK1 motif as was demonstrated for the Light-green_WGCNA _module (Additional File 3).

The Green-yellow_Diff _module, which was significantly co-expressed only for the low muscling trait, was weakly enriched for terms generally relating to *RNA processing*, *transcription factor activity and regulation*, and *ubiquitin-mediated protein catabolism *(Additional Files 7 and 9). This differentially expressed module was associated with the low muscling EBV trait and therefore it is suggested that this trait was primarily the result of both enhanced RNA processing and enhanced protein catabolism. GSEA predicted ten enriched promoter motifs of which six were known transcription factor binding motifs (Additional File 3). Of these transcription factors, PAX3 (paired box 3) was particularly noteworthy as it is a marker of resident myogenic progenitor cells [37]. This may indicate that PAX3 links muscle RNA and protein turnover with maintenance of myogenic progenitor cells in the low muscling trait. SREBP1 (sterol regulatory element-binding protein 1) was also identified in this analysis. It links control of muscle mass with lipid metabolic pathways, thus connecting muscle formation with energy regulation [38].

Analysis of the Salmon_Diff _module only revealed weak enrichment for terms summarised as *carbohydrate metabolism *and *RNA processing *(Additional File 7). GSEA revealed seven enriched transcription factor motifs and one anonymous motif (enrichment scores = 1.12 and 1.02, respectively) (Additional File 3). SREBP1 was again identified. Also of note was GATA6, which has been implicated as a negative regulator of cardiac muscle development [39].

The Orange_Diff _module showed enrichment for *regulation of transcription *and *protein catabolism *(Additional File 7). The latter term was consistent with the low muscling status in sire groups IV-VI. GSEA analysis revealed three anonymous motifs and seven enriched transcription factor motifs including motifs for C/EBP and MEF2A (Additional File 3). The latter, as mentioned above, is a pivotal promyogenic transcription factor while the former was also identified in the Violet_Diff _module.

While the WGCNA and differential co-expression network analyses are inherently different in their approaches, both yielded broadly similar conclusions in terms of the relationships between the sire groups and the functional term enrichments associated with identified modules. Higher resolution of the analyses could be anticipated by increasing the number of progeny per sire and the number of sire groups although there was surprisingly strong genetic architecture in the gene expression patterns in the current investigation given the restricted number of sires groups evaluated.

## Conclusions

There was strong genetic architecture to the gene expression program in skeletal muscle samples taken from the progeny of sires characterised by a range of EBVs for EMD (Figure 1). The genetic basis of population variation for most complex traits, like muscling, is thought to be the result of contributions from a large number of polymorphic genes each of small effect size. Within an animal many genes act by coordinate activation and repression of specific biological pathways to elicit cumulative effects on the muscling trait. Genetic variations in these genes are likely to contribute to variation in the trait within the population. The current gene expression network analyses identified pathways operating in skeletal muscle that are likely to contribute to quantitative trait variation in the trait EMD, a measure of muscle yield. Polymorphic variations in a subset of genes encoding proteins directly participating in these pathways, their regulation or in early developmental processes underpinning the formation of these pathways are likely to be responsible for genetic variation in the EMD trait.

Gene co-expression network analyses are more likely to identify modules containing co-regulated genes whose encoded proteins directly contribute to structural units such as muscle fibres, proteosomes, ribosomes and mitochondria, all of which were identified in the current analyses. Genes involved in regulating the formation of these structural units are likely to be a primary source of the genetic variation determining sire EBV status. The strong proteosome signature (Cyan_WGCNA _module) indicated that there was increased emphasis on protein degradation in the low muscling EBV groups. The enrichment for the NRF1 transcription factor binding motif in the promoters of genes in this module is consistent with the role for NRF1 in regulating 26 S proteosome formation [22]. Therefore, NRF1 and its related counterpart NFE2L1 are potential candidates for examination of genetic variation contributing to variation in the EMD trait. Regulators possibly contributing to the genetic variation in muscle fibre formation included *PPP2CA *(calcineurin) and *DICER1*, which were identified in the Violet_WGCNA _and Violet_Diff _modules. These genes are known to control muscle function and formation. Calcineurin has a key role in the hypertrophy response of skeletal muscle and DICER1 is essential for muscle formation as it catalyses the processing of pre-miRNA, some of which are essential for myogenesis [32-35,40].

**Figure 5:**
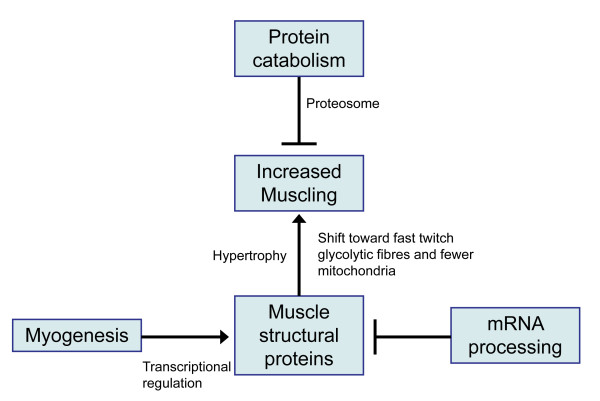
**Major pathways contributing to the genetics of the trait eye muscle depth**. The diagram summarises the major pathways implicated in the genetics of enhanced muscling in sheep longissimus lumborum skeletal muscle. Arrows denote feed forward effects while truncated lines denote repression.

Figure 5 summarises the major pathway contributions to the quantitative variation in the EMD trait. It is recognised that the model is likely to be an oversimplification. At the model's core is muscle accretion mediated by enhanced myogenesis, muscle fibre hypertrophy and decreased protein catabolism i.e. the key biological determinant dictating EBV status is the balance between muscle fibre protein accretion and turnover. An additional contribution includes mRNA processing. The differential gene expression network analysis suggests that the EMD trait is the result of different emphases on these functional pathways in the high and low muscling groups. For example, the high EBV status is more likely to be mediated by increased muscle protein formation and not by decreased muscle protein degradation, whereas low EBV status may primarily be the result of increased emphasis on protein degradation. By contrast, the enhanced muscling arising from the action of some hormonal growth promotants is predominately mediated by decreased protein degradation [41].

Enhanced muscling is probably also linked with a fibre type shift toward fast twitch glycolytic fibres, which have fewer mitochondria than slow twitch oxidative fibres. Consistent with this conclusion, direct examination of the longissimus lumborum muscle in the progeny of Poll Dorset sires characterised by high EBVs for EMD demonstrated an increased proportion of fast twitch glycolytic Type IIb fibres compared with progeny from sires with low EBVs for this trait, although the number of myofibres and overall size of myofibres for each fibre type were unaffected by EBV [42]. These results indicate that intense genetic selection for increased EMD in this sheep population is also likely to select for increased glycolytic fibres in the longissimus muscle. Whether this impacts on the overall growth performance of the animals through differing metabolic emphases in skeletal muscle is not known. The same study showed that total RNA and total protein were higher in the progeny of high muscling sires. This result is also consistent with the model shown in Figure 5.

Analysis of the promoters of genes in the identified modules often suggested that specific transcription factors provided multi-functional linkages between the processes of muscle formation, protein turnover and energy homeostasis. For example, the transcription factor NRF1 linked regulation of the formation of the energy dependent proteosome with mitochondrial biogenesis, while C/EBP linked muscle formation with adipose tissue formation and energy homeostasis. Thus, these highly integrated regulatory systems provide increased opportunity for feedback control mechanisms that maintain muscle mass within genetically predefined limits but also in a state that is responsive to the overall energy balance in the animal. This may be a consistent theme in the genetics underpinning variation in muscling. It is interesting to note that five of the enriched transcription factor motifs (for binding of NRF1, NFE2L1, RUNX1, TGIF1 and MEIS1) were also indentified in an analysis of bovine skeletal muscle development using gene expression information and a different analysis methodology [43].

The future challenge is to identify causal genetic polymorphisms contributing to variation in the EMD trait. The integration of high density SNP information with these network analyses, especially using highly connected hub genes, may accelerate the discovery of cis-acting genetic polymorphisms that alter gene expression and thereby directly contribute to this muscling trait [4,44].

## Methods

### Animals

Six Poll Dorset sires with a range of Estimated Breeding Values (EBVs) of accuracy greater than 90% for the yearling trait Eye Muscle Depth (EMD) were identified in Australian industry flocks using LAMBPLAN [45] (Table 1). These sires were crossed with merino ewes and large numbers of progeny were subsequently assessed for a number of performance traits. Sheep were bred from a research flock raised at University of New England (NSW, Australia). The EMD trait is correlated with muscle yield and is based on ultrasonic measurement of 'eye' muscle depth at the 'C' site (45 mm from the midline between the 12^th ^and 13^th ^rib) in longissimus lumborum muscle. The EBV for EMD measured the genetic difference (in mm) in the trait for a sire based on two seasons of yearling progeny data (2003-2004; 2004-2005) compared with contemporary sire groups set as a baseline in 2001. Typically, each sire was progeny tested using several hundred offspring. The high muscling sire group (sires I-III) represented sires with EBVs in the 1^st^-15^th ^percentiles. All other sires (sires IV-VI) were categorised into a lower muscling sire group where their EBVs were in the 45^th^-95^th ^percentiles. Although sire V had an EBV of +0.9 it was placed into the lower muscling category because of its percentile ranking (45^th^) relative to the rankings of sires in the high muscling group (1^st^-15^th ^percentiles). None of the animals carried the known myostatin or callipyge mutations, both of which result in enhanced muscling (results not shown).

### Biological samples

A total of 40 progeny (18 month old ewes) from the 6 sires (4-8 animals/sire) were identified and these were used for microarray analysis of skeletal muscle samples. Sheep were euthanized at commercial abattoirs for sample collection in accordance with the animal ethics guidelines of University of New England (NSW). A sample of longissimus lumborum muscle was dissected from each animal at the same pre-determined site within 15 minutes of euthanasia, snap frozen under liquid nitrogen and stored at -80°C until subsequent RNA extraction.

### RNA extraction and cDNA synthesis

Total RNA was extracted from 0.5 g of longissimus lumborum muscle from each animal by pulverisation under liquid nitrogen followed by extraction using Trizol reagent (Invitrogen). RNA (100 μg) for each sample was further purified by Dnase1 (Ambion) treatment followed by spin column purification by using an Rneasy Mini Kit (Qiagen) and on-column Dnase1 treatment. The RNA was quantified spectrophotometrically and its integrity verified by the OD_260_/OD_280 _absorption ratio (> 1.8) and by visualization on an agarose gel. cDNA synthesis was undertaken with 5 μg of isolated total RNA per sample using MMLV Superscript III reverse transcriptase (Invitrogen) and an anchored oligo-T_18 _primer combined with random hexamers [9].

### Microarray gene expression data

Microarray gene expression data were obtained by using the Affymetrix GeneChip^® ^Bovine Genome Array (Affymetrix, Santa Clara, CA), which contains 23,995 probe sets representing ~19,000 UniGene clusters (Bovine UniGene Build 57). Target labelling, hybridisations, fluidics and chip scanning to obtain an intensity value for each probe were performed as described elsewhere [9]. Microarray data have been deposited at the National Centre for Biotechnology Information (NCBI) Gene Expression Omnibus (GEO) (Accession number GSE20552). The microarray is deficient in full representation of myosin heavy chain genes, which are often used to define skeletal muscle fibre composition [7].

### Microarray data processing

Previous studies indicated that the bovine Affymetrix microarray can be used for analysis of ovine gene expression although there was some minor data loss due to species specific sequence differences in the probe sets [7-9]. Several probe set algorithms were available for processing microarray data. All of the algorithms, which include MAS5 (Microarray Suite), RMA (Robust Multichip Average), GC-RMA (Robust Multichip Average with adjustment for GC content of probes) and PLIER (Probe Logarithmic Intensity Error), have strengths and weaknesses [46,47]. Consequently, two primary microarray processing strategies were undertaken. The first method was a highly conservative analysis using MAS5 (Microarray Suite) in combination with its independent flag calls of *present*, *marginal *and *absent *i.e. only probe-sets with significant signal above background and having flag calls of *present *or *marginal *in all 40 microarrays were used. A detailed description of MAS5 analysis can be found elsewhere [7,9]. The signal for a specific probe-set was calculated from the weighted average of all probe signals in the probe set using One-step Tukey's Biweight estimates and summarized as log_2 _scaled averages. This approach has led to good correspondence between microarray and qRT-PCR analyses [7-9]. These probe sets were then filtered to include only those with MAS5 flag calls of *present *or *marginal *in all samples. These data, which consist of 5,394 genes, were used for hierarchical clustering analysis. MAS5 is less robust for detecting differentially expressed probes across treatments however it does not attempt to normalise data across microarrays. In this sense it is a more unbiased estimate of gene expression intensities and therefore more suitable for clustering of the entire dataset. The second method used GC-RMA for data processing, which was then used as input for subsequent gene network constructions. GC-RMA is more robust than MAS5 for identification of differentially expressed genes and it removes scatter at low intensity signals and accounts for differences in probe GC content. In this case, Mismatch intensities and the independent MAS5 flag calls were not used as this could have eliminated interesting genes that were expressed but in only a subset of animals. GC-RMA data analysis is described elsewhere [48]. Annotation of probe-sets was previously described [9] and since then has been augmented by multifaceted searches of several publically available resources based on the Btau 4.0 bovine genome assembly [49,50]. The term 'probe-set' was largely replaced by 'gene' in subsequent text. Some probes sets could not be uniquely annotated and were therefore not used in the functional enrichment analyses. Gene symbols are placed in italics except when used in the context of a protein.

### Unsupervised hierarchical clustering

Unsupervised hierarchical cluster analysis used as input MAS5 data filtered for the flag calls of *present *or *marginal *in all 40 microarrays. This combination is a highly conservative approach to data analysis. The data were analysed using GeneSpring GX software (Agilent Technologies) with selection for "condition tree" and "gene tree" with "Standard Correlation". Fischer's Exact test calculated in GeneSpring was used to determine the significance of the association between gene expression and sire group or sire EBV status.

### Gene expression network construction

GC-RMA was used for primary signal generation and normalisation for subsequent network analyses [48]. Probe sets whose mean expression levels were very low (log_2 _probe intensity < 2.35) or whose expression levels varied little (S.D. < 0.01) over the experimental samples were removed. This left 13,409 of 23,995 probe-sets. The exclusion of some probe-sets was undertaken on the basis that non-changing genes provide little information in a gene co-expression setting. All statistical analyses for network constructions were performed in the R statistical programming environment using the Bioconductor open source package [51,52]. In order to analyse the data within a weighted gene co-expression network analysis (WGCNA; [5]) framework in a reasonable time frame, the size of the data set was first reduced by selection based on connectivity, which is a measure of the number of genes showing pairwise co-expression with a specific gene. The connectivity of a particular gene is defined as the sum of the connection strengths between that particular gene and all other genes in the network. Genes with a high connectivity are thought to be biologically important as they reflect tightly regulated processes and when this connectivity changes these genes likely contribute to an altered phenotype. The 3,500 most highly connected genes in each of the high and low muscling datasets (giving a combined unique set of 5,223 genes) were selected for construction of gene co-expression networks.

Networks were created by using a component of the WGCNA framework of Langfelder and Horvath [5]. A summary of network terms and an example of application of the WGCNA framework can be found elsewhere [4]. First, the co-expression similarity was calculated from the expression values by using the Pearson correlation coefficient and then it was transformed into an adjacency matrix using a 'soft thresholding' approach which maintained the continuous nature of the similarity measure matrix [4]. The co-expression similarity was raised to a power β (soft thresholding) to maintain high similarity measures as high adjacencies but lower similarity measures were pushed towards an adjacency of zero. *Beta *was chosen based on the scale-free topology criterion [10] whereby the linear regression model fitting index (R^2^) was used to quantify how well the network satisfied this criterion. We chose β in the interval (1,15), which maximised the scale-free topology fit (R^2 ^≥ 0.8). To define clusters of genes in the data set, the adjacency matrix was used to calculate the topological overlap measure (TOM), which shows the degree of overlap in shared neighbours between pairs of genes in the network [14]. A topological overlap of 1 means that two genes share all the same neighbourhood-genes, while a topological overlap of 0 means that two genes don't share any neighbourhood genes. A TOM-plot visualized this relative interconnectedness between pairs of genes and showed how modules (clusters of highly interconnected genes) were created. Modules were defined using the Dynamic Tree Cutting algorithm [5,15] on a dendrogram created from the dissimilarity TOM matrix. Modules were required to initially have a minimum of 40 members and these were then used as input into the WGCNA and differential co-expression network analyses. Forty two modules were identified. The relative merits of these network approaches have been previously discussed [4,53].

### Weighted gene co-expression network analysis

The modules defined above were then selected on their module correlation (MC), which is the absolute correlation between the module eigengene (a representative gene expression of the module) and sire EBV for EMD. Only modules with MC greater than 0.4 were selected for further analysis. Genes within those modules were then retained in the modules based on their intra-modular connectivity and their correlation with the EBV status. The intra-modular connectivity is a single gene measure of connectivity within a module [10]. It is calculated by taking the correlation between a particular gene and the module eigengene. Thus, genes in selected modules were only retained in the selected modules if: (i) their intra-modular connectivity was > 0.6; (ii) their intra-modular connectivity with other modules was < 0.6, and; (iii) the absolute correlation of the gene expression with the EBV status was > 0.5. The retained genes in the identified modules were subsequently analysed by functional enrichment analysis (see below).

### Differential co-expression network

It was hypothesized that gene expression profiles of modules were up- or down-regulated in a manner that contributed to the muscling phenotype of the sire group. The coXpress R-package was used for differential co-expression analysis of the pre-defined modules [6]. The software uses a re-sampling method to calculate a P-value for each of the 42 predefined modules. Briefly, coXpress generated 10,000 data sets made up of modules of equal size to the original data set, but comprised of randomly assigned genes. For each module a null distribution was generated using the t-statistic calculated for each of the 10,000 replicates. The t-statistic of the original modules was then compared to the null distribution and a P-value was calculated to determine if it was significantly different from random (P < 0.05) or not different from random (P > 0.3). These t-statistics and P-values were calculated for both the low- and high-muscling EBV conditions. A module was then defined as differentially co-expressed when it was significantly different from random in one condition but not in the other condition. In these analyses the correlation of the genes with the muscling status was already taken into account and therefore genes in the identified modules were retained based on different criteria than for WGCNA. Genes in the differentially co-expressed modules were retained in the selected modules only if: (i) their intra-modular connectivity was > 0.6, and; (ii) their intra-modular connectivity with other modules was < 0.6. Only modules retaining 15 or more genes were then selected for functional enrichment analysis.

### Module names

All defined modules were assigned a name based on a colour. However, genes in the modules were retained based on different criteria in the two network analyses and for that reason the lists of genes in each analysis for the same module were not necessarily identical. To delineate this difference in the module gene content, a subscript was added to each module name. For the WGCNA analysis "WGCNA" was added as a subscript to the module name and for the differential co-expression network analysis "Diff" was added as a subscript.

### Functional enrichment analyses

Functional enrichment analysis was used to assign biological relevance to the gene network modules by using AgriGO [16,54]), the Database for Annotation, Visualization and Integrated Discovery (DAVID) [17,18,55] or Gene Set Enrichment Analysis (GSEA) [19,56]. In general, conservative default parameters were used in these analyses. The genes present on the Affymetrix microarray were used as the background for statistical analyses. All P-values were corrected for multiple testing using the Benjamini method. Unannotated probe sets were removed from analyses. These arise from incomplete annotation of the bovine genome sequence, non-unique gene assignments or incorrect probe-set construction. AgriGO was designed to provide visualisation of gene ontology (GO) information focussed on agricultural species by presentation of hierarchical tree graphs of overrepresented GO terms. The information inside the box for a significant term, included: gene ontology (GO) term; adjusted P-value; GO description; item number mapping the GO in the query list and background, and; total numbers for the query list and background. Boxes with smaller adjusted P-values were coloured darker and redder. Uncoloured boxes contained terms with adjusted P-values greater than 0.05. Solid, dashed, and dotted lines represent two, one and zero enriched terms at both ends connected by the line, respectively. DAVID provides a comprehensive set of functional annotation tools and statistical methods for identification of enriched biological terms within gene data sets. To obtain a higher level perspective of any term enrichments, *Functional Annotation Clustering *was performed in DAVID. This higher level analysis displayed similar functional annotations together based on overlaps of genes associated with each function term and therefore gives a clearer overview of gene function information associated with large datasets. A cluster enrichment score (- log (geometric mean of the P-values for terms in the cluster)) greater than 1.0 was considered significant. The conservative *High Stringency *option was used. Conserved gene-promoter associated anonymous motifs present in the human, mouse, rat and dog genomes and conserved gene-promoter associated transcription factor binding sites were identified using GSEA. The conserved anonymous motifs and transcription factor binding sites were restricted to a sequence window of ± 2 kb of the consensus transcription start site. Motifs are defined in the TRANSFAC (v. 7.4) database [57]. In some instances slightly different motifs for binding of the same transcription factor have been reported. The output from GSEA is an enrichment score, describing the imbalance in the distribution of ranks of gene expression in each gene set. The number of genes in the overlap (k) was set at ≥ 3. The enrichment score was normalized according to size of the gene sets which were then ranked according to the normalized enrichment score. The default False Positive Rate (FDR) *q*-value setting (FDR *q*-value < 0.25) was used as the cut-off.

### The potential influence of SNP positioned within probes sets

It is possible that SNP within probe sets confound interpretation of the gene expression data [58,59]. This possibility is unlikely to be a dominant influence for the following reasons. (i) The Affymetrix gene expression probe set for each gene consists of eleven 25 mers and thus SNP within a single oligonucleotide probe have minimal impact on the probe set signal. (ii) The software used for processing the microarray data typically deemphasizes outlier probes within the probe set and thus the resulting probe set signal is not sensitive to genetic variation. Moreover, there is strong correlation between bovine and ovine probe set signals for skeletal muscle samples indicating that even interspecies sequence variation does not affect the probe set signal for the majority of genes (> 75%) [9]. There is also strong correspondence between microarray probe set signals and qRT-PCR signals [7-9]. The latter analysis frequently used amplicons outside of the probe set region. (iii) The frequency of overlap of SNP within probe sets has been calculated for rodents and humans and shown to be a minor influence [59,60]. Moreover, direct investigations of probe sets by resequencing have revealed that the impact of probe based SNP on the detection of cis-acting QTLs is limited [61]. (iv) If SNP do overlap with probe sets then there is no a priori reason why there should be gene clustering according to EBV status for EMD or gene set enrichment corresponding to specific terms e.g. muscle fibre structural proteins. In the former case, the highly conservative *present *or *marginal *MAS5 flag call filter required for all 40 microarrays in the hierarchical clustering analysis shown in Figure 1 would have eliminated any probe sets adversely affected by SNP variation in the animal population and yet there was still strong genetic structure in the dataset.

## Authors' contributions

RT and HO conceived the original study linking gene expression with sire EBV status. TV and KB extracted RNA and performed the microarray analyses and hierarchical clustering analysis. CG and RT contributed to microarray data processing. LK, NW-H, HK designed and undertook the network analyses. LK and RT analysed the network modules and interpreted functional information. JK, HO, GG and RT identified animals and secured muscle samples. RT and LK wrote the manuscript. All authors read and approved the final version of the manuscript.

## Supplementary Material

Additional file 1 **Genes in the identified WGCNA modules and the differential gene co-expression network modules**. This file lists the genes present in the identified WGCNA modules and the differential gene co-expression network modules. Each module was identified by a colour name. The Affymetrix probe set, gene symbol and gene name are listed. Some probe sets could not be uniquely annotated. Click here for file

Additional file 2 **Functional annotation clustering of genes present in the identified WGCNA modules**. Summary table listing enriched functional annotation clusters and functional terms associated with genes in the identified WGCNA modules. The analysis was performed using DAVID analysis tools, which provide a higher order perspective of functional term enrichments as enriched clusters of terms [18]. Conservative parameters were used in the analysis. P-values were corrected for multiple testing using the Benjamini correction. The enrichment score for a Functional Annotation Cluster is the -log (geometric mean of the term P-values within the cluster). Enrichment scores ≥1.0 were considered significant. No enriched functional annotation clusters were identified for genes in the Violet_WGCNA _module using these parameters. Click here for file

Additional file 3 **Enriched motifs present in the promoters of genes in the identified WGCNA and differentially connected modules**. Genes present in each of the identified WGCNA and differentially connected modules were examined by using GSEA for enrichment of conserved cis-acting regulatory motifs [19]. The database within GSEA included gene-associated anonymous motifs conserved in the human, mouse, rat and dog genomes and conserved gene-associated transcription factor binding sites. The motifs were restricted to a 'promoter' sequence window corresponding to ± 2 kb of the transcription start site. P-values ≤ 0.05 were considered significant. Click here for file

Additional file 4 **Hierarchical tree graphs of over-represented GO terms for genes in the Lightgreen_WGCNA _module**. Hierarchical tree graphs of over-represented gene ontology (GO) terms for genes in the Lightgreen_WGCNA _module were constructed using AgriGO [16]. Boxes in the graphs represent GO terms labelled by GO number, term definition and statistical information. Significant terms (adjusted P ≤ 0.05) are coloured. The degree of colour saturation of a box is positively correlated to the enrichment level of the term. Solid, dashed, and dotted lines represent two, one and zero enriched terms at both ends connected by the line, respectively. GO categories: (a) Molecular Function; (b) Biological Process; (c) Cellular Component. Click here for file

Additional file 5 **Hierarchical tree graphs of over-represented GO terms for genes in the Violet_WGCNA _module**. Hierarchical tree graphs of over-represented gene ontology (GO) terms for genes in the Violet_WGCNA _module were constructed using AgriGO [16]. Boxes in the graphs represent GO terms labelled by GO number, term definition and statistical information. The analysis was performed using less stringent parameters (adjusted P < 0.1 and ≥ 2 genes/term) than the default parameters. Significant terms are coloured. The degree of colour saturation of a box is positively correlated to the enrichment level of the term. Solid, dashed, and dotted lines represent two, one and zero enriched terms at both ends connected by the line, respectively. GO categories: (a) Biological Process; (b) Cellular Component. Click here for file

Additional file 6 **Hierarchical tree graphs of over-represented GO terms for genes in the Violet_Diff _module**. Hierarchical tree graphs of over-represented gene ontology (GO) terms for genes in the Violet_Diff _module were constructed using AgriGO [16]. Boxes in the graph represent GO terms labelled by GO number, term definition and statistical information. The analysis was performed using default parameters. Significant terms are coloured (adjusted P ≤ 0.05). The degree of colour saturation of a box is positively correlated to the enrichment level of the term. Solid, dashed, and dotted lines represent two, one and zero enriched terms at both ends connected by the line, respectively. Only the Cellular Component GO category was significant. Click here for file

Additional file 7 **Functional annotation clustering of genes present in the differential gene co-expression modules**. Summary table listing enriched functional annotation clusters and functional terms significantly associated with the genes in the identified differential gene co-expression modules. The analysis was performed using DAVID analysis tools which provide a higher order perspective of functional term enrichments [18]. Conservative analysis parameters were used in the analysis. P-values were corrected for multiple testing using the Benjamini correction. The enrichment score for a Functional Annotation Cluster is the -log (geometric mean of the term P-values within the cluster). Enrichment scores ≥ 1.0 were considered significant. Click here for file

Additional file 8 **Hierarchical tree graphs of over-represented GO terms for genes in the Light-green_Diff _module**. Hierarchical tree graphs of over-represented gene ontology (GO) terms for genes in the Light-green_Diff _module were constructed using AgriGO [16]. Boxes in the graphs represent GO terms labelled by GO number, term definition and statistical information. The analysis was performed using default parameters. Significant terms are coloured (adjusted P ≤ 0.05). The degree of colour saturation of a box is positively correlated to the enrichment level of the term. Solid, dashed, and dotted lines represent two, one and zero enriched terms at both ends connected by the line, respectively. Click here for file

Additional file 9 **Hierarchical tree graphs of over-represented GO terms for genes in the Green-yellow_Diff _module**. Hierarchical tree graphs of over-represented gene ontology (GO) terms for genes in the Green-yellow_Diff _module were constructed using AgriGO [16]. Boxes in the graphs represent GO terms labelled by GO number, term definition and statistical information. The analysis was performed using default parameters. Significant terms are coloured (adjusted P ≤ 0.05). The degree of colour saturation of a box is positively correlated to the enrichment level of the term. Solid, dashed, and dotted lines represent two, one and zero enriched terms at both ends connected by the line, respectively. Only terms in the Biological Process GO category were significant. Click here for file

## References

[B1] Gilmour AR Luff AF Fogarty NM Banks R Genetic-Parameters for Ultrasound Fat Depth and Eye Muscle Measurements in Live Poll Dorset Sheep Australian Journal of Agricultural Research 1994 45 6 1281 1291 10.1071/AR9941281

[B2] Rockman MV Kruglyak L Genetics of global gene expression Nature reviews 2006 7 11 862 872 10.1038/nrg1964 17047685

[B3] Ponsuksili S Murani E Schwerin M Schellander K Wimmers K Identification of expression QTL (eQTL) of genes expressed in porcine M. longissimus dorsi and associated with meat quality traits BMC genomics 11 572 10.1186/1471-2164-11-572 PMC3091721 20950486

[B4] Kadarmideen HN Watson-Haigh NS Andronicos NM Systems biology of ovine intestinal parasite resistance: disease gene modules and biomarkers Molecular Biosystems 2011 7 1 235 246 10.1039/c0mb00190b 21072409

[B5] Langfelder P Horvath S WGCNA: an R package for weighted correlation network analysis BMC bioinformatics 2008 9 559 10.1186/1471-2105-9-559 19114008 PMC2631488

[B6] Watson M CoXpress: differential co-expression in gene expression data BMC bioinformatics 2006 7 509 10.1186/1471-2105-7-509 17116249 PMC1660556

[B7] Byrne K Vuocolo T Gondro C White JD Cockett NE Hadfield T Bidwell CA Waddell JN Tellam RL A gene network switch enhances the oxidative capacity of ovine skeletal muscle during late fetal development BMC genomics 2010 11 378 10.1186/1471-2164-11-378 20546621 PMC2894804

[B8] Fleming-Waddell JN Wilson LM Olbricht GR Vuocolo T Byrne K Craig BA Tellam RL Cockett NE Bidwell CA Analysis of gene expression during the onset of muscle hypertrophy in callipyge lambs Animal genetics 2007 38 1 28 36 10.1111/j.1365-2052.2006.01562.x 17257185

[B9] Vuocolo T Byrne K White J McWilliam S Reverter A Cockett NE Tellam RL Identification of a gene network contributing to hypertrophy in callipyge skeletal muscle Physiological genomics 2007 28 3 253 272 17077277 10.1152/physiolgenomics.00121.2006

[B10] Zhang B Horvath S A general framework for weighted gene co-expression network analysis Statistical applications in genetics and molecular biology 2005 4 Article17 10.2202/1544-6115.1128 16646834

[B11] Farber CR Identification of a Gene Module Associated With BMD Through the Integration of Network Analysis and Genome-Wide Association Data Journal of Bone and Mineral Research 2010 25 11 2359 2367 10.1002/jbmr.138 20499364

[B12] MacLennan NK Dong J Aten JE Horvath S Rahib L Ornelas L Dipple KM McCabe ERB Weighted gene co-expression network analysis identifies biomarkers in glycerol kinase deficient mice Molecular Genetics and Metabolism 2009 98 1-2 203 214 10.1016/j.ymgme.2009.05.004 19546021

[B13] Mason MJ Fan GP Plath K Zhou Q Horvath S Signed weighted gene co-expression network analysis of transcriptional regulation in murine embryonic stem cells BMC genomics 2009 10 10.1186/1471-2164-10-327 PMC2727539 19619308

[B14] Ravasz E Somera AL Mongru DA Oltvai ZN Barabasi AL Hierarchical organization of modularity in metabolic networks Science (New York, NY) 2002 297 5586 1551 1555 10.1126/science.1073374 12202830

[B15] Langfelder P Zhang B Horvath S Defining clusters from a hierarchical cluster tree: the Dynamic Tree Cut package for R Bioinformatics (Oxford, England) 2008 24 5 719 720 10.1093/bioinformatics/btm563 18024473

[B16] Du Z Zhou X Ling Y Zhang Z Su Z agriGO: a GO analysis toolkit for the agricultural community Nucleic acids research 2010 38Web Server W64 70 10.1093/nar/gkq310 PMC2896167 20435677

[B17] Huang da W Sherman BT Lempicki RA Bioinformatics enrichment tools: paths toward the comprehensive functional analysis of large gene lists Nucleic acids research 2009 37 1 1 13 10.1093/nar/gkn923 19033363 PMC2615629

[B18] Huang da W Sherman BT Lempicki RA Systematic and integrative analysis of large gene lists using DAVID bioinformatics resources Nature protocols 2009 4 1 44 57 19131956 10.1038/nprot.2008.211

[B19] Subramanian A Tamayo P Mootha VK Mukherjee S Ebert BL Gillette MA Paulovich A Pomeroy SL Golub TR Lander ES Gene set enrichment analysis: a knowledge-based approach for interpreting genome-wide expression profiles Proceedings of the National Academy of Sciences of the United States of America 2005 102 43 15545 15550 10.1073/pnas.0506580102 16199517 PMC1239896

[B20] Oddy VH Herd RM McDonagh MB Woodgate R Quinn CA Zirkler K Effect of divergent selection for yearling growth rate on protein metabolism in hind-limb muscle and whole body of Angus cattle Livestock Production Science 1998 56 3 225 231 10.1016/S0301-6226(98)00153-5

[B21] Ramachandran B Yu G Gulick T Nuclear respiratory factor 1 controls myocyte enhancer factor 2 A transcription to provide a mechanism for coordinate expression of respiratory chain subunits The Journal of biological chemistry 2008 283 18 11935 11946 10.1074/jbc.M707389200 18222924 PMC2335360

[B22] Steffen J Seeger M Koch A Kruger E Proteasomal degradation is transcriptionally controlled by TCF11 via an ERAD-dependent feedback loop Molecular cell 40 1 147 158 10.1016/j.molcel.2010.09.012 20932482

[B23] MacIntosch BR Gardiner PF McComas AJ Skeletal muscle form and function Human Kinetics 2006

[B24] Virbasius JV Scarpulla RC Activation of the human mitochondrial transcription factor A gene by nuclear respiratory factors: a potential regulatory link between nuclear and mitochondrial gene expression in organelle biogenesis Proceedings of the National Academy of Sciences of the United States of America 1994 91 4 1309 1313 10.1073/pnas.91.4.1309 8108407 PMC43147

[B25] Yoshida T Gan Q Franke AS Ho R Zhang J Chen YE Hayashi M Majesky MW Somlyo AV Owens GK Smooth and cardiac muscle-selective knock-out of Kruppel-like factor 4 causes postnatal death and growth retardation The Journal of biological chemistry 2008 285 27 21175 21184 10.1074/jbc.M110.112482 PMC2898332 20439457

[B26] Yoshida T Gan Q Owens GK Kruppel-like factor 4, Elk-1, and histone deacetylases cooperatively suppress smooth muscle cell differentiation markers in response to oxidized phospholipids American journal of physiology 2008 295 5 C1175 1182 10.1152/ajpcell.00288.2008 18768922 PMC2584997

[B27] Grade CV Salerno MS Schubert FR Dietrich S Alvares LE An evolutionarily conserved Myostatin proximal promoter/enhancer confers basal levels of transcription and spatial specificity in vivo Development genes and evolution 2009 219 9-10 497 508 10.1007/s00427-009-0312-x 20052486

[B28] Heidt AB Rojas A Harris IS Black BL Determinants of myogenic specificity within MyoD are required for noncanonical E box binding Molecular and cellular biology 2007 27 16 5910 5920 10.1128/MCB.01700-06 17562853 PMC1952131

[B29] Wang X Blagden C Fan J Nowak SJ Taniuchi I Littman DR Burden SJ Runx1 prevents wasting, myofibrillar disorganization, and autophagy of skeletal muscle Genes & development 2005 19 14 1715 1722 10.1101/gad.1318305 16024660 PMC1176009

[B30] Benders AA Veerkamp JH Oosterhof A Jongen PJ Bindels RJ Smit LM Busch HF Wevers RA Ca2+ homeostasis in Brody's disease. A study in skeletal muscle and cultured muscle cells and the effects of dantrolene an verapamil The Journal of clinical investigation 1994 94 2 741 748 10.1172/JCI117393 8040329 PMC296154

[B31] Rossi AE Dirksen RT Sarcoplasmic reticulum: the dynamic calcium governor of muscle Muscle & nerve 2006 33 6 715 731 10.1002/mus.20512 16477617

[B32] Mallinson J Meissner J Chang KC Chapter 2. Calcineurin signaling and the slow oxidative skeletal muscle fiber type International review of cell and molecular biology 2009 277 67 101 19766967 10.1016/S1937-6448(09)77002-9

[B33] Ge Y Chen J MicroRNAs in skeletal myogenesis Cell cycle (Georgetown, Tex) 2011 10 3 441 448 10.4161/cc.10.3.14710 PMC3115018 21270519

[B34] Wong CF Tellam RL MicroRNA-26a targets the histone methyltransferase Enhancer of Zeste homolog 2 during myogenesis The Journal of biological chemistry 2008 283 15 9836 9843 10.1074/jbc.M709614200 18281287

[B35] O'Rourke JR Georges SA Seay HR Tapscott SJ McManus MT Goldhamer DJ Swanson MS Harfe BD Essential role for Dicer during skeletal muscle development Developmental biology 2007 311 2 359 368 10.1016/j.ydbio.2007.08.032 17936265 PMC2753295

[B36] Wu Z Rosen ED Brun R Hauser S Adelmant G Troy AE McKeon C Darlington GJ Spiegelman BM Cross-regulation of C/EBP alpha and PPAR gamma controls the transcriptional pathway of adipogenesis and insulin sensitivity Molecular cell 1999 3 2 151 158 10.1016/S1097-2765(00)80306-8 10078198

[B37] Yokoyama S Asahara H The myogenic transcriptional network Cell Mol Life Sci10.1007/s00018-011-0629-2 PMC3092062 21318263

[B38] Lecomte V Meugnier E Euthine V Durand C Freyssenet D Nemoz G Rome S Vidal H Lefai E A new role for sterol regulatory element binding protein 1 transcription factors in the regulation of muscle mass and muscle cell differentiation Molecular and cellular biology 2010 30 5 1182 1198 10.1128/MCB.00690-09 20028734 PMC2820883

[B39] Xin M Davis CA Molkentin JD Lien CL Duncan SA Richardson JA Olson EN A threshold of GATA4 and GATA6 expression is required for cardiovascular development Proceedings of the National Academy of Sciences of the United States of America 2006 103 30 11189 11194 10.1073/pnas.0604604103 16847256 PMC1544063

[B40] Michel RN Dunn SE Chin ER Calcineurin and skeletal muscle growth The Proceedings of the Nutrition Society 2004 63 2 341 349 10.1079/PNS2004362 15294053

[B41] Sinnett-Smith PA Dumelow NW Buttery PJ Effects of trenbolone acetate and zeranol on protein metabolism in male castrate and female lambs The British journal of nutrition 1983 50 2 225 234 10.1079/BJN19830092 6193805

[B42] Greenwood PLDJJ Gaunt GM Ferrier GR Influences on the loin and cellular characteristics of the m. longissimus lumborum of Australian Poll Dorset-sired lambs Australian Journal of Agricultural Research 2006 57 1 12 10.1071/AR04316

[B43] Gu Q Nagaraj SH Hudson NJ Dalrymple BP Reverter A Genome-wide patterns of promoter sharing and co-expression in bovine skeletal muscle BMC genomics 12 23 10.1186/1471-2164-12-23 PMC3025955 21226902

[B44] Kadarmideen HN Genetical systems biology in livestock: application to gonadotrophin releasing hormone and reproduction IET systems biology 2008 2 6 423 441 10.1049/iet-syb:20070072 19045837

[B45] LAMBPLAN http://www.sheepgenetics.org.au/LAMBPLAN/Default.aspx

[B46] Pepper SD Saunders EK Edwards LE Wilson CL Miller CJ The utility of MAS5 expression summary and detection call algorithms BMC bioinformatics 2007 8 273 10.1186/1471-2105-8-273 17663764 PMC1950098

[B47] Seo J Gordish-Dressman H Hoffman EP An interactive power analysis tool for microarray hypothesis testing and generation Bioinformatics (Oxford, England) 2006 22 7 808 814 10.1093/bioinformatics/btk052 16418236

[B48] Wu ZJ Irizarry RA Preprocessing of oligonucleotide array data Nature Biotechnology 2004 22 6 656 658 15175677 10.1038/nbt0604-656b

[B49] Elsik CG Tellam RL Worley KC Gibbs RA Muzny DM Weinstock GM Adelson DL Eichler EE Elnitski L Guigo R The genome sequence of taurine cattle: a window to ruminant biology and evolution Science (New York, NY) 2009 324 5926 522 528 10.1126/science.1169588 PMC2943200 19390049

[B50] Tellam RL Lemay DG Van Tassell CP Lewin HA Worley KC Elsik CG Unlocking the bovine genome BMC genomics 2009 10 193 10.1186/1471-2164-10-193 19393070 PMC2680899

[B51] Bioconductor http://bioconductor.org/

[B52] Gentleman RC Carey VJ Bates DM Bolstad B Dettling M Dudoit S Ellis B Gautier L Ge Y Gentry J Bioconductor: open software development for computational biology and bioinformatics Genome biology 2004 5 10 R80 10.1186/gb-2004-5-10-r80 15461798 PMC545600

[B53] de la Fuente A From 'differential expression' to 'differential networking' - identification of dysfunctional regulatory networks in diseases Trends Genet 2010 26 7 326 333 10.1016/j.tig.2010.05.001 20570387

[B54] AgriGO http://bioinfo.cau.edu.cn/agriGO/

[B55] Database for Annotation, Visualization and Integrated Discovery (DAVID) http://david.abcc.ncifcrf.gov PMC193660 12734009

[B56] Gene Set Enrichment Analysis http://www.broadinstitute.org/gsea/index.jsp

[B57] TRANSFAC - Transcription Factor Binding Predictions http://biobase-international.com/index.php?id=transfac

[B58] Alberts R Terpstra P Li Y Breitling R Nap JP Jansen RC Sequence polymorphisms cause many false cis eQTLs PloS one 2007 2 7 e622 10.1371/journal.pone.0000622 17637838 PMC1906859

[B59] Doss S Schadt EE Drake TA Lusis AJ Cis-acting expression quantitative trait loci in mice Genome research 2005 15 5 681 691 10.1101/gr.3216905 15837804 PMC1088296

[B60] Alberts R Terpstra P Hardonk M Bystrykh LV de Haan G Breitling R Nap JP Jansen RC A verification protocol for the probe sequences of Affymetrix genome arrays reveals high probe accuracy for studies in mouse, human and rat BMC bioinformatics 2007 8 132 10.1186/1471-2105-8-132 17448222 PMC1865557

[B61] de Koning DJ Haley CS Genetical genomics in humans and model organisms Trends Genet 2005 21 7 377 381 10.1016/j.tig.2005.05.004 15908034

