# Supplementary figures and images for: Genetic architecture of gene expression in ovine skeletal muscle
Source: BMC Genomics. 2011 Dec 15;12:607. doi: 10.1186/1471-2164-12-607 (PMC3265547; doi:10.1186/1471-2164-12-607)

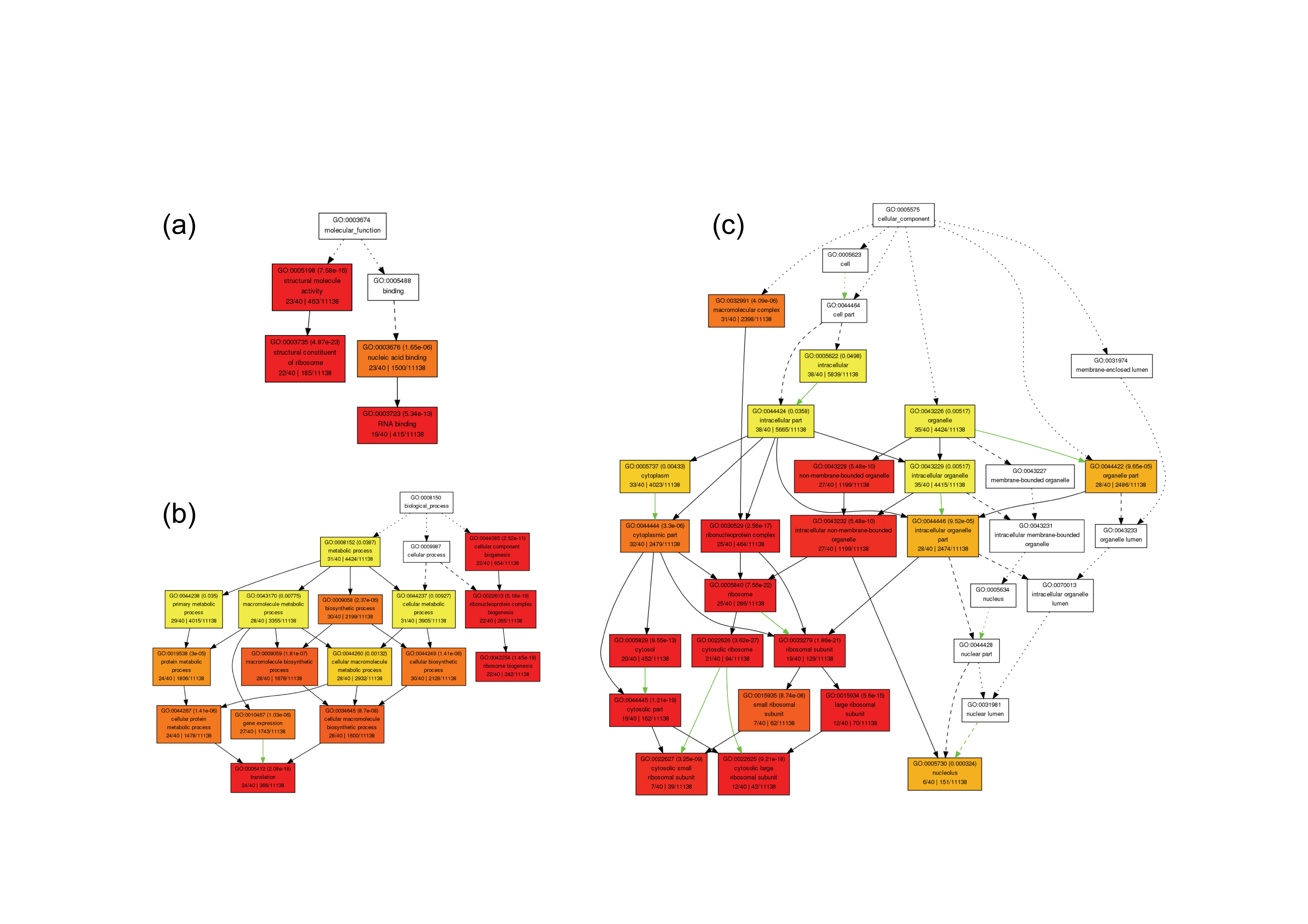

Supplement: Additional file 4 — Hierarchical tree graphs of over-represented GO terms for genes in the LightgreenWGCNA module. Hierarchical tree graphs of over-represented gene ontology (GO) terms for genes in the LightgreenWGCNA module were constructed using AgriGO [16]. Boxes in the graphs represent GO terms labelled by GO number, term definition and statistical information. Significant terms (adjusted P ≤ 0.05) are coloured. The degree of colour saturation of a box is positively correlated to the enrichment level of the term. Solid, dashed, and dotted lines represent two, one and zero enriched terms at both ends connected by the line, respectively. GO categories: (a) Molecular Function; (b) Biological Process; (c) Cellular Component. [file 1471-2164-12-607-S4.TIFF]

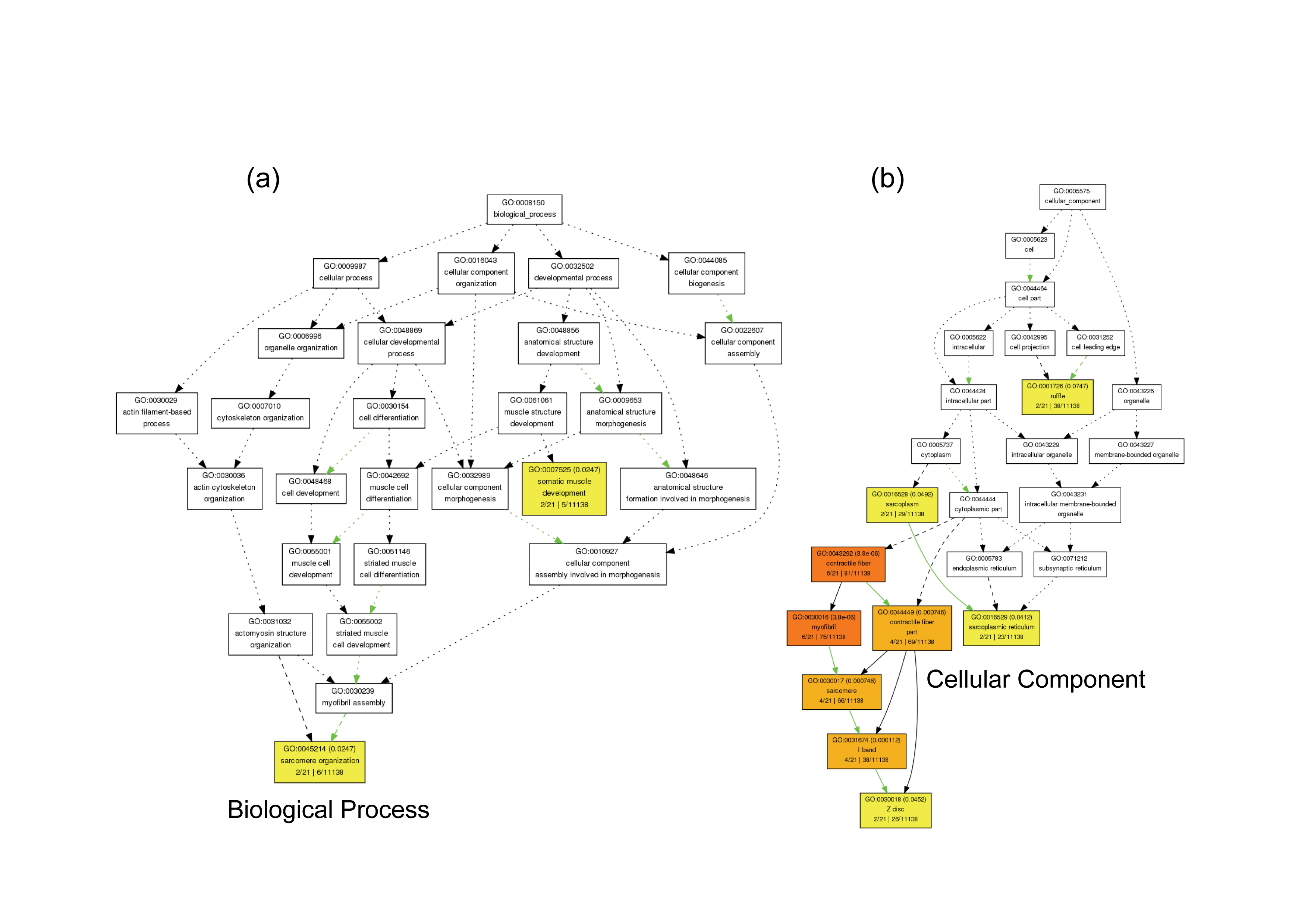

Supplement: Additional file 5 — Hierarchical tree graphs of over-represented GO terms for genes in the VioletWGCNA module. Hierarchical tree graphs of over-represented gene ontology (GO) terms for genes in the VioletWGCNA module were constructed using AgriGO [16]. Boxes in the graphs represent GO terms labelled by GO number, term definition and statistical information. The analysis was performed using less stringent parameters (adjusted P < 0.1 and ≥ 2 genes/term) than the default parameters. Significant terms are coloured. The degree of colour saturation of a box is positively correlated to the enrichment level of the term. Solid, dashed, and dotted lines represent two, one and zero enriched terms at both ends connected by the line, respectively. GO categories: (a) Biological Process; (b) Cellular Component. [file 1471-2164-12-607-S5.TIFF]

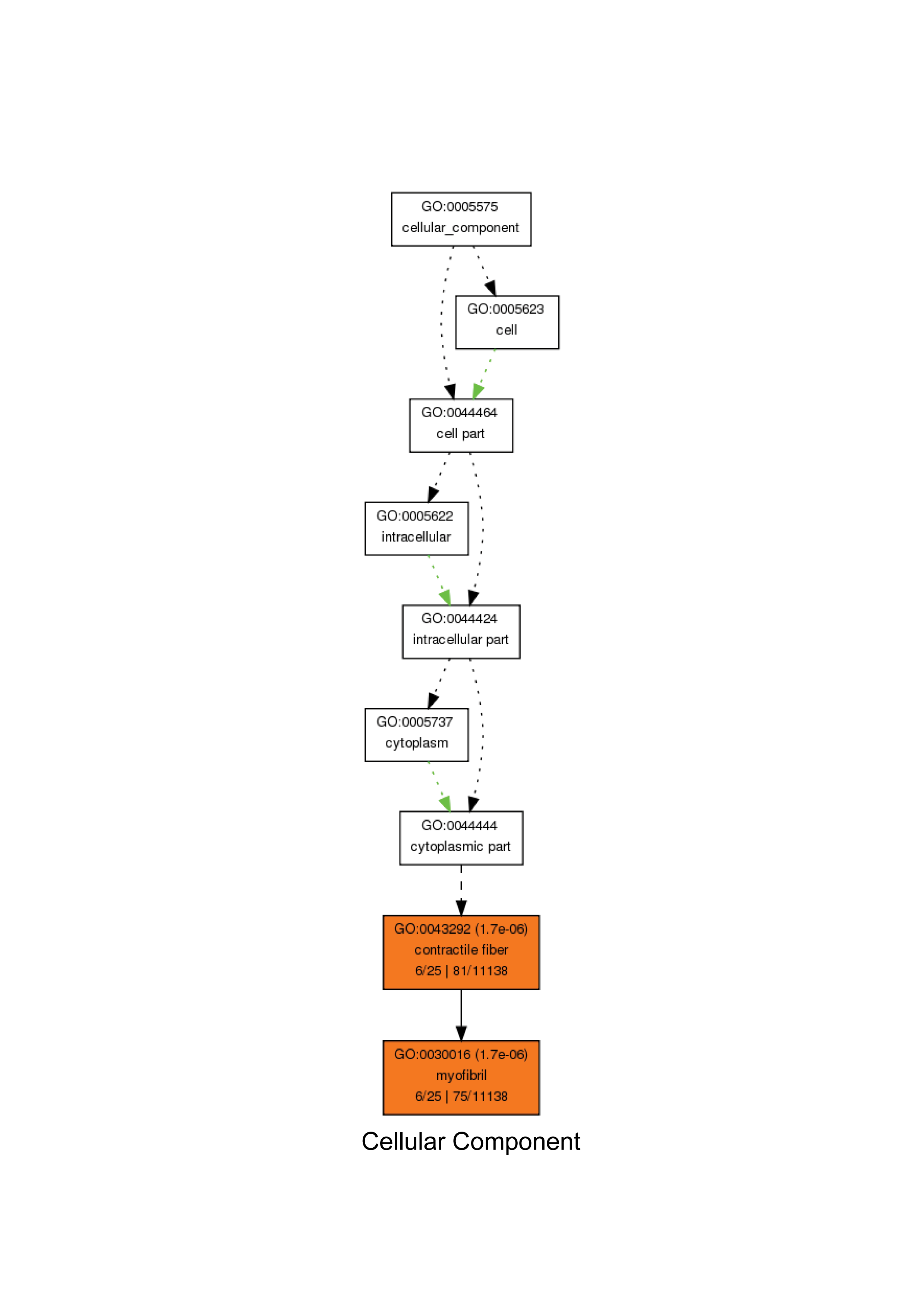

Supplement: Additional file 6 — Hierarchical tree graphs of over-represented GO terms for genes in the VioletDiff module. Hierarchical tree graphs of over-represented gene ontology (GO) terms for genes in the VioletDiff module were constructed using AgriGO [16]. Boxes in the graph represent GO terms labelled by GO number, term definition and statistical information. The analysis was performed using default parameters. Significant terms are coloured (adjusted P ≤ 0.05). The degree of colour saturation of a box is positively correlated to the enrichment level of the term. Solid, dashed, and dotted lines represent two, one and zero enriched terms at both ends connected by the line, respectively. Only the Cellular Component GO category was significant. [file 1471-2164-12-607-S6.TIFF]

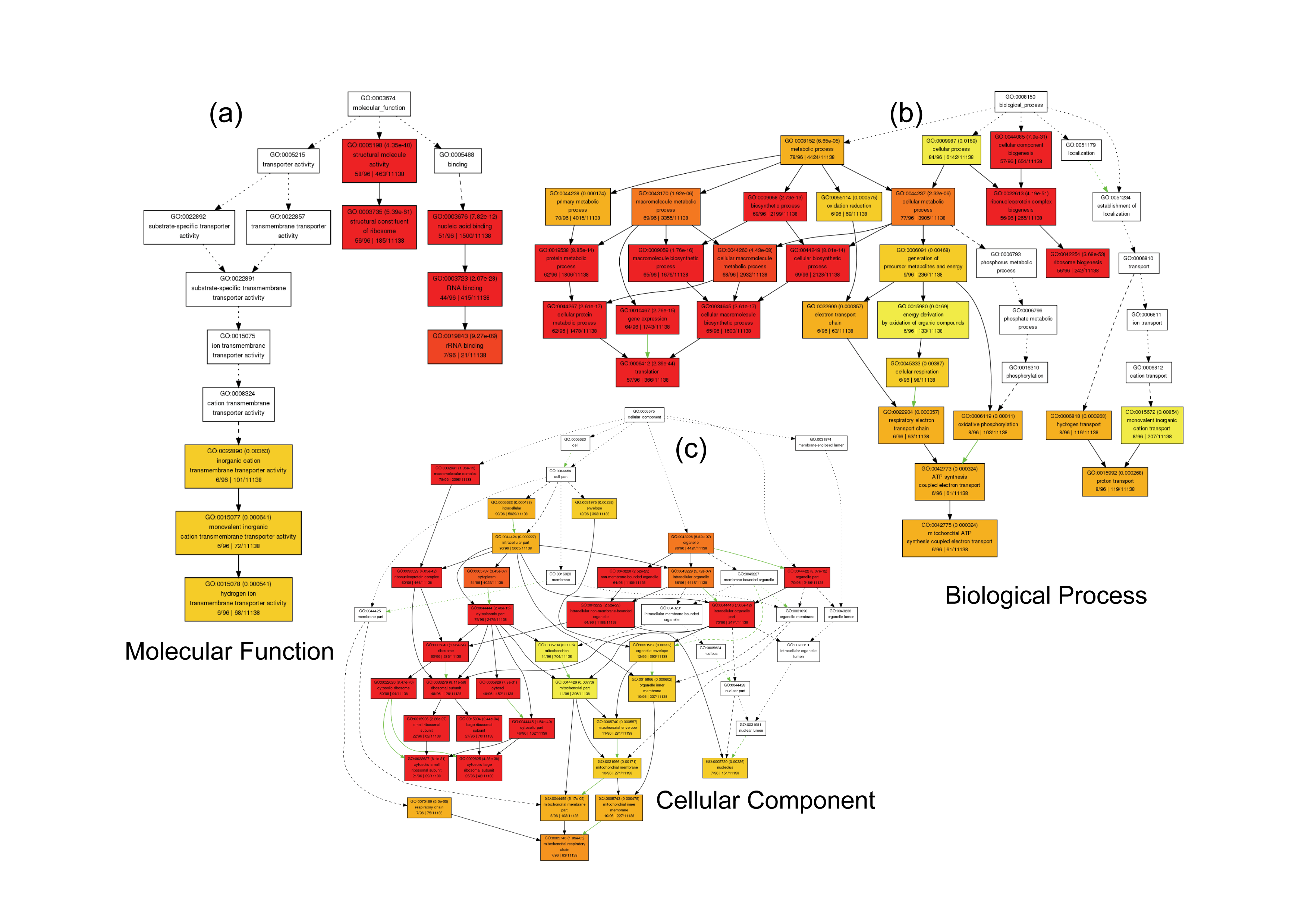

Supplement: Additional file 8 — Hierarchical tree graphs of over-represented GO terms for genes in the Light-greenDiff module. Hierarchical tree graphs of over-represented gene ontology (GO) terms for genes in the Light-greenDiff module were constructed using AgriGO [16]. Boxes in the graphs represent GO terms labelled by GO number, term definition and statistical information. The analysis was performed using default parameters. Significant terms are coloured (adjusted P ≤ 0.05). The degree of colour saturation of a box is positively correlated to the enrichment level of the term. Solid, dashed, and dotted lines represent two, one and zero enriched terms at both ends connected by the line, respectively. [file 1471-2164-12-607-S8.TIFF]

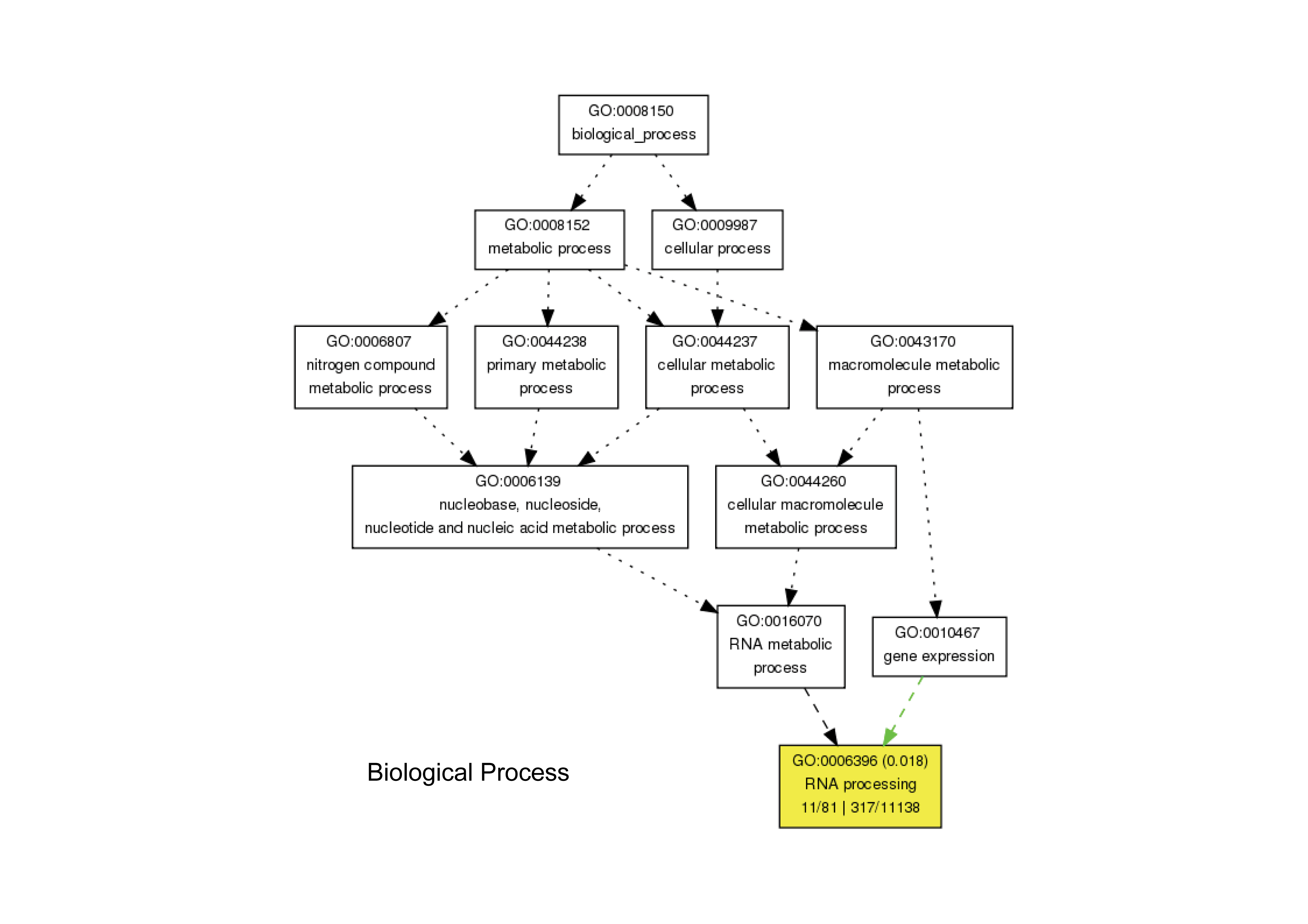

Supplement: Additional file 9 — Hierarchical tree graphs of over-represented GO terms for genes in the Green-yellowDiff module. Hierarchical tree graphs of over-represented gene ontology (GO) terms for genes in the Green-yellowDiff module were constructed using AgriGO [16]. Boxes in the graphs represent GO terms labelled by GO number, term definition and statistical information. The analysis was performed using default parameters. Significant terms are coloured (adjusted P ≤ 0.05). The degree of colour saturation of a box is positively correlated to the enrichment level of the term. Solid, dashed, and dotted lines represent two, one and zero enriched terms at both ends connected by the line, respectively. Only terms in the Biological Process GO category were significant. [file 1471-2164-12-607-S9.TIFF]
